# What are the working mechanisms of a web-based workplace sitting intervention targeting psychosocial factors and action planning?

**DOI:** 10.1186/s12889-017-4325-5

**Published:** 2017-05-03

**Authors:** Katrien De Cocker, Ilse De Bourdeaudhuij, Greet Cardon, Corneel Vandelanotte

**Affiliations:** 1 Department of Movement and Sports Sciences, Ghent University, Watersportlaan 2, B-9000 Ghent, Belgium; 2 Research Foundation Flanders, Egmonstraat 5, B-1000 Brussels, Belgium; 3 Physical Activity Research Group, School for Health, Medical and Applied Sciences, Central Queensland University, Bruce Highway, North Rockhampton, QLD 4702 Australia

**Keywords:** Sedentary behaviour, Computer-tailoring, Employees, E-health, Mediation analyses

## Abstract

**Background:**

Office workers demonstrate high levels of sitting on workdays. As sitting is positively associated with adverse health risks in adults, a theory-driven web-based computer-tailored intervention to influence workplace sitting, named ‘Start to Stand,’ was developed. The intervention was found to be effective in reducing self-reported workplace sitting among Flemish employees. The aim of this study was to investigate through which mechanisms the web-based computer-tailored intervention influenced self-reported workplace sitting.

**Methods:**

Employees (*n* = 155) participated in a clustered randomised controlled trial and reported socio-demographics (age, gender, education), work-related (hours at work, employment duration), health-related (weight and height, workplace sitting and physical activity) and psychosocial (knowledge, attitudes, self-efficacy, social support, intention regarding (changing) sitting behaviours) variables at baseline and 1-month follow-up. The product-of-coefficients test of MacKinnon based on multiple linear regression analyses was conducted to examine the mediating role of five psychosocial factors (knowledge, attitudes, self-efficacy, social support, intention). The influence of one self-regulation skill (action planning) in the association between the intervention and self-reported workplace sitting time was investigated via moderation analyses.

**Results:**

The intervention had a positive influence on knowledge (*p* = 0.040), but none of the psychosocial variables did mediate the intervention effect on self-reported workplace sitting. Action planning was found to be a significant moderator (*p* < 0.001) as the decrease in self-reported workplace sitting only occurred in the group completing an action plan.

**Conclusions:**

Future interventions aimed at reducing employees’ workplace sitting are suggested to focus on self-regulatory skills and promote action planning when using web-based computer-tailored advice.

**Trial registration:**

Clinicaltrials.gov NCT02672215; (Archived by WebCite at https://clinicaltrials.gov/ct2/show/NCT02672215).

**Electronic supplementary material:**

The online version of this article (doi:10.1186/s12889-017-4325-5) contains supplementary material, which is available to authorized users.

## Background

Web-based computer-tailored interventions have been found to be feasible, acceptable, and successful in changing a variety of health-related behaviours, including alcohol consumption, smoking habits, dietary behaviours, and physical activity [1–4]. These types of interventions have the ability to use a personalized approach while reaching large numbers of participants, resulting in easy to implement and low cost interventions. However, few web-based computer-tailored interventions have targeted sedentary behaviours (i.e. activities in a sitting or reclining posture characterized by a low energy expenditure) [5] which have been identified as important independent health-risk factors. Both the duration (total amount of sitting) and pattern (prolonged sitting bouts) of sedentary behaviours have been linked to increased risks of chronic diseases such as obesity, metabolic syndrome, type 2 diabetes, some cancers, and cardio-vascular disease and all-cause mortality [6–10]. Levels of sedentary behaviour are high, especially in the workplace, where 71–77% of working hours are being spent sedentary [11–13]. As such, workplace interventions should aim to reduce and interrupt workplace sitting time.

A web-based, computer-tailored intervention, Start to Stand [14], including constructs based on the Theory of Planned Behaviour (TPB) [15] and aspects of the Self-Regulation Theory (SRT) [16] was recently developed. After completing an assessment questionnaire, users received personalized computer-tailored feedback about their sitting time and tips on how to change their sitting. A feasibility and acceptability study was conducted among mostly female (74.9%), highly educated (71.3%) employees from a public city service (*n* = 112; mean age: 41.0 ± 9.5 years). This theory-driven intervention was found to be acceptable in terms of the assessments, attractiveness, length, credibility and relevance of the advice [14]. In addition, a clustered randomized controlled trial was conducted to examine the intervention effect on self-reported sitting outcomes (at work, during transport, during TV viewing, during computer use, and other leisure sitting) among University and environmental agency employees (*n* = 155). Objectively determined (via ActivPAL™) sitting outcomes (percentage of sitting at work, standing at work, and number of breaks at work) were additionally assessed in a subsample (*n* = 108) [17]. The web-based, tailored intervention was effective in reducing self-reported workplace sitting among Flemish employees [17]: in the intervention condition self-reported workplace sitting decreased (−59 min/day) after 1 month compared to a generic (non-tailored advice) and control (no advice) condition. However, it should be noted that this effect was not confirmed by the objective data assessed in the subsample. At this stage, the mechanisms through which this significant effect on self-reported workplace sitting was generated remain unknown. It is unknown whether targeting the psychosocial factors on which the advice was tailored (knowledge, attitudes, self-efficacy, social support, intention), or whether promoting a self-regulation skill (action planning), or a combination were responsible for the positive intervention effect. Thus to increase knowledge on the working mechanisms of the web-based, computer-tailored intervention, it is important to investigate the variables that contributed to its effectiveness in changing workplace sitting.

Few studies have examined the working mechanisms of computer-tailored interventions. One study on the mediating variables of a web-based computer-tailored nutrition education intervention in adults found that only some of the targeted determinants (intention, food availability, attitude, subjective norm) mediated the effect of the intervention on dietary intake [18], while others (e.g. awareness, self-efficacy, action planning, coping planning) did not influence the intervention effect, depending on the dietary behaviour. In a computer-tailored weight prevention intervention for overweight adults, psychological factors such as self-regulation skill action planning were associated with repeated use of the intervention [19]. Studies examining the working mechanisms of computer-tailored interventions targeting sedentary behaviour seem to be lacking. However, there is some evidence linking theoretical constructs with sedentary behaviour. In the study of Prapavessis et al. [20], factors consistent with the TPB explained 9–58% and 8–43% of the variance in intention and sedentary behaviour respectively, revealing the growing evidence that the TPB is a useful framework for understanding sedentary behaviour. Furthermore, a recent review of behaviour change strategies used in sedentary behaviour reduction interventions among adults revealed that among other things interventions based on education and self-regulatory skills were promising [21].

Therefore, the overall objective of this paper was to investigate through which mechanisms the web-based computer-tailored intervention [14, 17] influenced self-reported workplace sitting in a sample of Flemish desk-based employees. The aims were to determine if the intervention had an influence on five psychosocial variables (knowledge, attitudes, self-efficacy, social support, intention) on which the advice was tailored, if these constructs mediated the effect of the intervention on workplace sitting time and if action planning was a moderator of the intervention.

## Methods

The methods are described below, however more detailed information about the study protocol is given elsewhere [17]. The study was registered in a Clinical Trial registry (Clinicaltrials.gov ID: NCT02672215) and the study protocols were approved by the Ethics Committee of the Ghent University Hospital, Belgium. All data can be find in Additional file 1 and the questions used in this study are presented in Additional file 2.

### Study design

The present study used data from a clustered randomized controlled trial with three different conditions (computer-tailored advice, generic advice, or control) conducted between October 2014 and March 2015. The present study utilized the self-reported data collected at pre-testing (T0) and 1-month follow-up (T1). There were also objective data available from a subsample, however it was preferable to use the self-reported data of the whole sample in order to increase power. Self-reported workplace sitting time was used as the present outcome, also because only for this measure a significant intervention effect was found in the total sample.

### Participants

Participants were employees from a convenience sample of two companies (university and environmental agency) in Flanders (i.e. northern Dutch-speaking part of Belgium), mainly employing desk-based workers, having more than 100 staff members and each having at least three different worksite locations. Within the university, three departments of the central administration were selected to participate and within the environment agency, three departments in East-Flanders were selected. Within each company, each department was randomly assigned (simple randomization) to one of three conditions (computer-tailored advice, generic advice, or control). These departments were selected using convenience sampling and because of their different physical locations, assuming little face-to-face contact between employees from the different departments, reducing the opportunity for contamination between groups.

### Recruitment and procedures

**Fig. 1:**
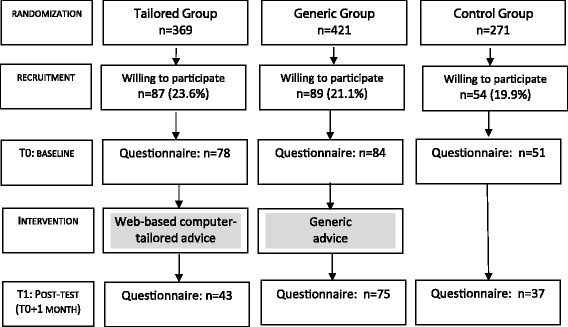
Diagram with flow of participants

Employees were invited to participate by e-mail (*n* = 1061) (see Fig. 1). No inclusion or exclusion criteria were used. A researcher e-mailed a confidential website username and password to all employees who agreed to participate (*n* = 230, response rate: 21.7%). After logging-in, which was done by 213 employees, participants were invited to complete an assessment questionnaire (see section Measures of the online questionnaire). At 1-month follow-up, 155 employees (72.8%) again completed the online questionnaire.

After completing the baseline questionnaire, each group received different feedback. The ‘tailored’ group (*n* = 78) received web-based computer-tailored advice including personalised feedback and tips on how to reduce and/or interrupt workplace sitting (see section Interventions). The ‘generic’ group (*n* = 84) received web-based advice containing generic information and tips to reduce and/or interrupt workplace sitting, which were not tailored to personal characteristics. The control group (*n* = 51) was a waitlist control condition and received the generic intervention after completing all measurements.

### Interventions

#### Computer-tailored intervention

The development of this theory-driven intervention has already been described in detail elsewhere [14, 17]. In brief, users received computer-tailored advice on their sitting time and suggestions on how to interrupt (having short standing breaks) and reduce (replacing sitting by periods of standing) sitting, after completing an assessment questionnaire. The questions, asked in order to provide the personal advice, obtained job-related information, knowledge about sedentary behaviour, and constructs of the TPB [15] including attitudes, self-efficacy, social norm and intention. The average time to complete the questionnaire was 16.3 min. A set of pre-defined decision rules selected the feedback messages that were matched and tailored to the specific answers given by the users during the initial assessment. These feedback messages appeared immediately on the user’s screen after completing the assessment questionnaire.

At the end of this tailored advice (‘section 1’), users were able to request up to 5 other non-committal specific sections if they were interested. All additional sections were available at once, but could be accessed at a later time. The structure and content of the assessment questionnaires (questions on sitting behaviours and its psychosocial factors including attitudes, self-efficacy, social support, perceived benefits and barriers, intention) and the advice (tailored feedback and tips to change the behaviour) itself of the additional sections were similar to the first section of the advice [14]. The focus was respectively on standing breaks during working hours (section 2a), replacing sitting by standing during working hours (section 2b), sitting during commuting to work (section 2c), sitting during (lunch) breaks at work (section 2d), and on making an action plan to improve sitting behaviour (section 2e). In section 2e, users motivated to change their sitting were invited to create an action plan through SMART (Specific, Measurable, Attainable, Relevant and Time-bound) goals and implementation-intentions [16, 22]. Users were asked *what* (increase standing breaks, or replace sitting by standing, or both) they wanted to do, *how long* (breaks: 10 s, 20 s, …, 4 min, 5 min; standing: 15 min, 30 min, …, 3 h 45 min, 4 h, >4 h), *how often* (breaks*:* every 5, 10, …, 55, 60 min*)*, and *when* (during working hours, lunch, commuting, or combinations) they wanted to change. Finally, users were asked *how* they wanted to change by selecting pre-composed ‘if-then’-statements (for example: When the phone rings, I will stand up; When I get a coffee, I will drink it while standing; When I get back at the office after lunch, I will work some time standing) or formulating new ‘if-then’-statements themselves (open-ended). When all questions were completed, a schematic overview of this personalized action plan appeared immediately on the user’s screen, and participants were able to print it.

#### Comparison conditions

The tailored condition was compared to a ‘generic advice’ condition and a control condition. In the generic advice, users received non-tailored information on the importance of reducing and interrupting sedentary behaviour and non-tailored suggestions on how to interrupt (having short standing breaks, 6 tips) and reduce (replacing sitting by periods of standing, 8 tips) sitting during work hours, (lunch) breaks, and commuting. Users were not able to make an action plan. The control condition did initially receive no information, but got the generic intervention after completing all measurements (i.e. wait-list control condition). In the present study, for ease of interpreting the study outcomes, the two conditions not receiving the tailored advice (generic and control) - which were also not effective in changing workplace sitting [17] - were collapsed into one comparison group, which has also been done elsewhere [23]. Results based on the analyses with the original generic and control group not being pooled (data not shown) did not differ from the current analyses presented in the paper.

### Measures of the online questionnaire

#### Outcome variable: Self-reported workplace sitting at 1-month follow-up

The level of workplace sitting time was assessed using two items from the Workforce Sitting Questionnaire (WSQ) [24] in which participants self-reported the time spent sitting while being at work on work and non-workdays. The WSQ has acceptable reliability (Intraclass Correlation Coefficient (ICC) = 0.63) and validity against objectively measured sitting time (*r* = 0.45) [24]. Values of workplace sitting over 12 h/day were truncated to 12 h to avoid unrealistic values [14].

#### Potential mediators: Psychosocial factors and action planning at 1-month follow-up

Five psychosocial factors, to which the advice was tailored to, were used as potential mediators in the present study (see Table 1). Participants’ *knowledge* about sedentary behaviour was asked using 3 items. *Attitudes* towards changing sitting were measured using 6 items. *Self-efficacy* was measured by asking how certain employees were about changing their sitting (4 items). *Social support* was assessed by asking whether colleagues would support them when trying to change their sitting behaviour. Finally, employees’ *intention* to change sitting was asked (used as continuous scale, see Table 1). All previous questions were based on previously validated questions to measure psychosocial correlates of physical activity [25]. The wording of the original questions was changed to reflect psychosocial correlates of sitting [26, 27]. All items were (re) coded into the same direction so that the highest scores were the most positive answers on each item. Cronbach’s α coefficients of internal consistency were calculated for attitudes and self-efficacy prior to computing the related items into one scale (see Table 1).

**Table 1:** Items and answering options of the psychosocial factors

Psychosocial factor	Items	Answering options	Internal consistency
Knowledge	1. ‘Prolonged daily sitting for long hours increases the risk of physical and mental health problems, like diabetes and depression’. 2. ‘Even when one is being regularly active, i.e. daily walking, prolonged sitting increases the risk of physical and mental health problems’. 3. ‘It is healthy to interrupt periods of prolonged sitting, as the risk of health problem then decreases’.	- disagree - unsure - agree	/
Attitudes	I think changing my sitting behaviour is… 1. … healthy. 2. … feasible. 3. … disturbing to others. 4. … awkward. 5. … relaxing. 6. … time-losing.	5-point scale ranging from ‘strongly disagree’ to ‘strongly agree’	α = 0.70
Self-efficacy	How certain are you about changing your sitting behaviour when… 1. … feeling tired/bad/tense/depressive? 2. … when colleagues don’t do this? 3. … when not being supported by your supervisors? 4. … being busy or having high time pressures?	5-point scale ranging from ‘strongly disagree’ to ‘strongly agree’	α = 0.81
Social support	Would your colleagues support you when trying to change your sitting behaviour?	5-point scale ranging from ‘strongly disagree’ to ‘strongly agree’	/
Intention	Are you intending to change your sitting behaviour?	- No - Yes, I may do this in the future - Yes, I will try this in the next weeks - Yes, I will start doing this right away	/

In addition to the psychosocial factors, the self-regulation skill, *action planning*, was considered as moderator of the intervention effect on self-reported workplace sitting. Whether or not participants completed the action planning section of the advice was collected from the website administration.

#### Covariates: Socio-demographic, work-related, health-related variables at baseline

Participants self-reported their age, gender, education [low (no diploma, elementary school, secondary school) vs high (high school, university)], average amount of time daily spent at work (hours-minutes), employment duration (number of years) and body mass and stature at baseline. Body mass index (BMI) was calculated with the following formula: body mass/stature^2^. The validated International Physical Activity Questionnaire (IPAQ) short version [28, 29] was used to assess the number of days and duration of time spent in walking, moderate intensity physical activity and vigorous intensity physical activity in the last week at baseline. Based on the guidelines for data processing and analysis of the IPAQ [30], total scores for walking, moderate and vigorous physical activities were computed (‘number of days’ x ‘duration of time’). Finally, the cluster variable (2 companies) was included as covariate in all the models.

### Data analyses

Analyses were conducted in 2016, using SPSS for Windows version 22.0 (SPSS Inc., Chicago, IL, USA). Due to the skewed nature of the outcomes, the analyses were performed on square root transformations to improve normality, but for reasons of clarity, non-transformed average scores were reported in the tables. To examine the mediating effect of the psychosocial variables, associations were tested using multiple linear regression models, controlled for all predefined covariates (age, gender, education, time at work, employment duration, BMI, time spent in walking, moderate physical activity, vigorous physical activity and clustering) and baseline values of the respectively outcome and independent variables in the regression. The bootstrapping approach was used to estimate 95% confidence intervals of the coefficients [31]. In the first stage of the analyses, the main association between the intervention and self-reported workplace sitting was tested (τ-coefficient). Due to the skewed nature of the outcome, analyses were done on the square root-transformations to improve normality [17]. In the second stage, the mediating role of the psychosocial factors on the association between the intervention and self-reported workplace sitting was tested using the product of-coefficients test of MacKinnon [31]. This test consists of four different steps. In the first step, the action theory tests estimate the association between the intervention and the potential mediators (α-coefficients). This is followed by the conceptual theory tests (step 2) which estimate the association between the potential mediators and the self-reported workplace sitting outcome (β-coefficients). In this step also τ’-coefficients (association between the intervention and the self-reported workplace sitting outcome, controlled for the mediator) are calculated. In step 3, the product of the two coefficients (αβ) was calculated, representing the mediating effect. In step 4, single mediation models (i.e. separately for each potential mediator) were conducted in which the coefficient αβ was divided by its standard error (SE) to assess the statistical significance of the mediating role (t-value). For the calculation of the SE, the Sobel test was used [SE_αβ_ = √(α^2^ SE_β_^2^ + β^2^ SE_α_^2^)] [32]. The obtained value of αβ/SEαβ was then compared to a standard normal distribution to report on the magnitude of p (z-values >1.960, >2.576, or >3.291 indicates a significant mediation effect at the 5% level, 1% level, and 0.1% level respectively) [29]. Furthermore, in case of significant mediation, the proportion mediating the association between the intervention and self-reported workplace sitting was estimated by dividing the indirect effect by the total effect, so the product of coefficients (αβ) was divided by the sum of αβ and the τ’-coefficient (αβ + τ’), resulting in the following formula: αβ/(αβ + τ’) * 100%.

To test the moderating effect of action planning, a repeated measures ANOVA test (within-subjects factor: time; between-subjects factor: condition with 3 levels including intervention group completing an action planning, intervention group without action planning, comparison group) was conducted. To increase power of the moderation analyses, intention-to-treat analyses (last value carried forward) were conducted.

## Results

### Participants’ characteristics and website usage

The total sample completing the online questionnaire at baseline (*n* = 213) consisted mainly of employees with a high education (82.1%), a white collar job (92% clerk, 8% management) and an employment duration of more than 5 years (69.8%). These participants (31.5% men) had a mean age of 40.3 ± 9.1 years, worked on average 8.0 ± 0.7 h/day and had a mean BMI of 23.9 ± 3.4 kg/m^2^. The baseline values of the outcome variable, potential mediators and covariates for both groups are presented in Table 2. No significant differences were found between both groups, except for the time spent sitting at work that was higher in the intervention group compared to the comparison group (see Table 1).

**Table 2:** Baseline values for the outcome variables, potential mediators and covariates of the study groups

	Web-based computer-tailored intervention (*n* = 78)	Comparison group (*n* = 135)
Outcome variable		
Workplace sitting: mean ± SD minutes/day	339.1 ± 125.1	284.6 ± 61.3^***^
Potential mediators		
Knowledge: mean ± SD	2.5 ± 0.3	2.6 ± 0.4
Attitudes: mean ± SD	3.9 ± 0.5	3.9 ± 0.6
Self-efficacy: mean ± SD	3.6 ± 0.8	3.6 ± 0.8
Social support: mean ± SD	1.9 ± 1.0	1.8 ± 1.0
Intention: n (%) intending to change	77/78 (98.7)	135/135 (100)
Action planning: n (%) completed	54/78 (69.2)	/
Covariates		
Age: mean ± SD years	40.4 ± 8.7	40.4 ± 9.7
Gender: n (%) men	26/78 (33.3)	42/135 (31.1)
Education: n (%) high school /university	59/78 (75.6)	116/135 (85.9)
Hours at work per day: mean ± SD	8.0 ± 0.9	8.0 ± 0.6
Employment duration: n (%) > 5 years	56/78 (71.8)	93/135 (68.9)
BMI: mean ± SD kg/m^2^	24.3 ± 3.1	23.7 ± 3.5
Walking: mean ± SD minutes/day	23.6 ± 31.9	25.3 ± 23.5
Moderate-intensity PA: mean ± SD minutes/day	31.0 ± 33.8	23.7 ± 24.8
Vigorous-intensity PA: mean ± SD minutes/day	10.4 ± 14.5	13.6 ± 19.7

*SD*: standard deviation, *PA* physical activity

^***^ *p* < 0.001

Website administration showed that a total of 66/78 employees completed section 2a (84.6%), 64/78 completed section 2b (82.1%), 60/78 completed section 2c (76.9%), 59/78 completed section 2d (75.6%), and 54/78 completed an action plan in section 2e (69.2%) at baseline. Of the 78 intervention participants at baseline, 43 remained in the study at 1-month follow-up and 41 of them had completed an action plan at baseline.

### Mediation analyses of the psychosocial variables

The intervention was significantly associated with workplace sitting at 1-month follow-up (see Main association test in Table 3) showing that receiving the computer-tailored advice was associated with less self-reported workplace sitting (*p* = 0.002). The intervention also had a positive influence on knowledge (*p* = 0.040) (see Action theory tests in Table 3). There was no significant influence of the intervention on attitudes, self-efficacy, social support and intention to change at 1-month follow-up (see Action theory tests in Table 3).

**Table 3:** Main association test, action theory tests and conceptual theory tests^a^

Main association test: association between intervention and workplace sitting			
	τ (SE)	95% CI	*p*
	−1.105 (0.376)	−1.871, −0.404^*^	0.002
Action theory tests: association between intervention and potential mediators			
Potential mediators	α (SE)	95% CI	*p*
Knowledge	0.132 (0.062)	0.006, 0.252^*^	0.040
Attitudes	0.030 (0.083)	−0.112, 0.197	0.724
Self-efficacy	−0.044 (0.112)	−0.259, 0.176	0.680
Social support	0.230 (0.192)	−0.152, 0.622	0.231
Intention	−0.325 (0.174)	−0.667, 0.023	0.059
Conceptual theory tests: association between potential mediators and workplace sitting			
Potential mediators	β (SE)	95% CI	*p*
Knowledge	−0.327 (0.469)	−1.262, 0.599	0.466
Attitudes	−0.569 (0.340)	−1.263, 0.040	0.097
Self-efficacy	0.044 (0.237)	−0.443, 0.503	0.845
Social support	−0.100 (0.165)	−0.417, 0.196	0.563
Intention	0.091 (0.217)	−0.384, 0.497	0.657

*CI* confidence interval

^a^adjusted for the cluster variable, age, gender, education, work hours, work duration, BMI, walking, moderate physical activity, vigorous physical activity, respective baseline value; coefficients results from analyses on the transformed sitting outcome

^*^ *p* < 0.05

The potential mediators were then included individually as additional predictors in the model that examined the association between the intervention and workplace sitting at 1-month follow-up (see Conceptual theory tests in Table 3). The potential mediators had no significant influence on the workplace sitting outcome (see Table 3).

Results of the mediation analyses are presented in Table 4. Knowledge, attitudes, self-efficacy, social support and intention did not significantly mediate the intervention effect on workplace sitting (see Table 4).

**Table 4:** Mediating role of psychosocial variables on the association between intervention and workplace sitting (single mediation models)^a^

Potential mediators	αβ (SE)	95% CI
Knowledge	−0.044 (0.066)	−0.173, 0.085
Attitudes	−0.017 (0.048)	−0.111, 0.077
Self-efficacy	−0.002 (0.012)	−0.026, 0.022
Social support	−0.023 (0.043)	−0.107, 0.061
Intention	−0.030 (0.072)	−0.171, 0.111

*CI* confidence interval

^a^adjusted for the cluster variable, age, gender, education, work hours, work duration, BMI, walking, moderate physical activity, vigorous physical activity, respective baseline value

### Moderation analyses of action planning

The intervention including the completing of an action plan was successful in decreasing self-reported workplace sitting (time x group: F = 14.3, *p* < 0.001), while this was not the case in the intervention condition not completing an action plan and in the comparison group having no intervention (see Table 5).

**Table 5:** Moderation effect of action planning

	Pre	1-month FU	F _time x group_ (*P*)^a^
Workplace sitting (mean ± SD minutes/day)			14.3 (0.001)
Intervention Group Completing Action Plan (*n* = 53)	332.8 ± 124.6	282.5 ± 105.3	
Intervention Group Without Action Planning (*n* = 23)	353.5 ± 128.1	351.8 ± 128.4	
Comparison Group (*n* = 124)	284.6 ± 61.3	283.5 ± 60.4	

^a^adjusted for the cluster variable, age, gender, education, work hours, work duration, BMI, walking, moderate physical activity, vigorous physical activity

## Discussion

The present study aimed to explore the mechanisms that may explain the intervention effect of the computer-tailored advice ‘Start to stand’ among Flemish employees. The intervention was previously found to be successful in reducing self-reported workplace sitting [17]. The current results showed that this intervention effect was moderated by action planning, though we were not able to identify mediating variables. This suggests that the intervention only resulted in lower levels of self-reported workplace sitting when an action plan had been completed. A recent review [21] also suggested to consider self-regulatory techniques for reducing sedentary behaviour. Action planning may be so important in sitting because of the habitual nature of this behaviour. The present finding on action planning is in line with the self-regulation theory, which postulates that skills like action planning are important for successful behaviour change as it targets pre- and post-intentional processes [16]. As a result, future programs aiming to reduce workplace sitting might encourage completing one or more action plans, however, the effect of action planning in itself still needs to be confirmed in randomized controlled trials (having intervention groups with or without access to action planning). Intention to change was high in all employees in the intervention group, and this was reflected in the number of employees (69.2% of the intervention participants) completing an action plan, which is in line with the theory. This number is very high compared to other computer-tailored interventions (respectively on multiple health behaviours, weight management, and smoking cessation) promoting action planning in which the planning tools were used by 17.8% of the participants for fruit and vegetable intake and 30.4% for physical activity [33], 15.9% for physical activity and 54.9% for dietary intake [19], and 30.3–89.4% (depending on the specific action, such as finding a buddy or removing ashtrays) [34]. In addition, it should be noted that in the present study, we only had information on whether or not the action plan was completed. In future research, it may be interesting to investigate the quality of the action planning and the degree in which the users followed-up on the plans. Plaete et al. for example found that health goal attainment in adults was predicted by the specificity of the implementation intentions, by the motivational value of the action plan and by making a new action plan at follow-up [35]. However, in the current study the link between the completion of the action plan and the level of engagement with the intervention in general is unknown. Nevertheless, post-hoc analyses did not show baseline differences between those completing and those not completing the action plan in age, gender, education, hours at work, employment duration, BMI, level of physical activity, knowledge, attitude, self-efficacy, social support and intention to change (data not shown). However, the majority of the intervention participants who completed an action plan (75.9%) completed the 1-month follow-up test, while the majority of those who did not complete an action plan (94.7%) dropped-out at 1-month.

Other studies examining the underlying mechanisms of a computer-tailored intervention targeting sitting could not be found. However, working mechanisms of some previous computer-tailored interventions targeting other health-behaviours were investigated. Findings on the influence of action planning in other computer-tailored interventions seem mixed. Some findings of web-based computer-tailored interventions for other health behaviours (weight gain prevention, fruit and vegetable consumption, physical activity) are in line with the present study in which action planning was also found to play a significant role in computer-tailored advice for workplace sitting [19, 33]. However, in another web-based computer-tailored nutrition education intervention, action planning was not found to be of significant influence on the intervention effects [18]. The inconsistent findings on action planning in computer-tailored interventions may be explained by the different health-behaviours that were targeted. However, studies examining the influence of action planning among non-tailored interventions show more consistent results on action planning and confirm the present finding [23, 36, 37]. But again sedentary behaviour was not the outcome, so until now limited evidence has been available for the mechanisms changing sedentary behaviour.

Psychosocial factors (knowledge, attitudes, self-efficacy, social support, intention) on which the advice was tailored were presently not found to be significant mediators of the intervention effect on workplace sitting. Results showed that the intervention did not influence these psychosocial factors (except for knowledge), while it was assumed that attitudes towards changing sitting, self-efficacy towards changing sitting, social support to change sitting time and the intention to change sitting would have increased after receiving the advice, as the tailoring was based on these variables. Among Canadian adults however, significant relationships were found between sedentary behaviour and intentions, attitudes, social norm and perceived behavioural control [20]. A potential explanation for the present lack of intervention effects on most of the psychosocial variables can be the high baseline values of these potential mediators, which was also found in some other computer-tailored interventions targeting several health behaviours [38–40]. Here, scores for attitudes and self-efficacy were above average (between 3.6 and 3.9 on a 5-point scale), which may indicate that the present study sample had already positive attitudes and a high self-efficacy at baseline, suggesting a ceiling effect. Furthermore, all employees in both the intervention and comparison groups already had the intention to change their sitting, so assessing positive changes in this variable was not possible. Only for social support, baseline scores seemed rather low (between 1.8 and 1.9 on a 5-point scale), but the present computer-tailored intervention was not able to change this.

Regarding the mediating effect of psychosocial factors in computer-tailored interventions targeting other health-behaviours (nutrition education, fruit and vegetable consumption, physical activity), most findings on attitude, self-efficacy and intention are in contrast to the present study [18, 37]. The present findings also seem to contrast findings of non-tailored interventions investigating the psychosocial factors as mediators, revealing that intention had a mediating role on the intervention effect on physical activity [23] and on exercise behaviour [37], and that self-efficacy mediated intervention effects on physical activity [36]. As previously indicated, no other studies investigated the mechanisms of interventions changing workplace sitting, so more research is needed on this topic.

A main strength of this study is that it is the first to examine the underlying mechanisms of a computer-tailored intervention targeting sitting time among employees. It should however be taken into account that the majority of the study sample consisted of highly educated, female, normal-weight employees with positive attitudes and high self-efficacy towards changing their sitting, and being motivated to change this behaviour. These aspects may limit generalizability of the present results. At baseline, the intervention group was more sedentary at work than the comparison group, which could mean they had more room for improvement. However, all analyses were controlled for this. Further, the present data, including the sitting outcome, were self-reported, which could have resulted in recall biases or social desirable answers. For example, employees who completed the action plan may have been more likely to believe that they had reduced their workplace sitting compared to those who did not complete an action plan, which may overestimate the impact of the moderating effect found. It should also be noted that no significant main interventions effects were found in the subsample providing objective measures of sitting. Further, there was a substantial drop-out rate in the intervention group (44.9%) and the majority of those dropping-out (62.9%) did not complete an action plan, so this might have influenced the findings as well. However intention-to-treat analyses were conducted to also take into account the effects among those not remaining in the study. Next, ceiling effects (attitudes, self-efficacy, intention) and lack of specificity (knowledge) might have occurred for the psychosocial factors. Finally, the long term impact of action planning and the effect of the intervention as a whole is unknown, as this study only used 1-month follow-up data. As a result, the present findings need to be confirmed in a long-term trial assessing objective sitting outcomes among a larger sample of various employees.

## Conclusions

The present analyses showed that only action planning was an effective intervention strategy used in the web-based computer-tailored advice aiming to reduce workplace sitting. The psychosocial factors targeted in the intervention were not affected by the advice and accordingly not found to be significant mediators of the intervention. More studies are needed to confirm the present findings, though at this stage it seems that future programs aiming to reduce workplace sitting might benefit from focusing on self-regulation skills, such as completing an action plan. More studies are also needed in order to examine the influence of psychosocial factors and action planning in interventions targeting other domains of sitting and other population groups.

## Additional files

Additional file 1: Dataset. Data collected and used in this study. (XLS 108 kb)

Additional file 2: Questionnaire. Overview of the questions used in this study. (DOCX 14 kb)

## Abbreviations

BMI: body mass index
CI: confidence interval
ICC: intraclass correlation coefficient
IPAQ: International Physical Activity Questionnaire
SD: standard deviation
SE: standard error
SMART: specific, measurable, attainable, relevant and time-bound
SRT: Self-Regulation Theory
TPB: Theory of Planned Behaviour
WSQ: Workforce Sitting Questionnaire

## References

[1] Kroeze W, Werkman A, Brug J (2006). A systematic review of randomized trials on the effectiveness of computer-tailored education on physical activity and dietary behaviors. Ann Behav Med.

[2] Neville LM, Milat AJ, O'Hara B (2009). Computer-tailored weight reduction interventions targeting adults: a narrative systematic review. Health Promot J Austr.

[3] Lustria ML, Cortese J, Noar SM, Glueckauf RL (2009). Computer-tailored health interventions delivered over the web: review and analysis of key components. Patient Educ Couns.

[4] Broekhuizen K, Kroeze W, van Poppel MN, Oenema A, Brug J (2012). A systematic review of randomized controlled trials on the effectiveness of computer-tailored physical activity and dietary behavior promotion programs: an update. Ann Behav Med.

[5] Sedentary Behaviour Research Network (2012). Letter to the editor: standardized use of the terms “sedentary” and “sedentary behaviours”. Appl Physiol Nutr Metab.

[6] Healy GN, Dunstan DW, Salmon J, Cerin E, Shaw JE, Zimmet PZ, Owen N (2008). Breaks in sedentary time: beneficial associations with metabolic risk. Diabetes Care.

[7] Proper K, Singh A, van Mechelen W, Chinapaw M (2011). Sedentary behaviors and health outcomes among adults. A systematic review of prospective studies. Am J Prev Med.

[8] Thorp A, Owen N, Neuhaus M, Dunstan D (2011). Sedentary behaviors and subsequent health outcomes in adults. A systematic review of longitudinal studies, 1996–2011. Am J Prev Med.

[9] Healy G, Matthews C, Dunstan D, Winkler E, Owen N (2011). Sedentary time and cardio-metabolic biomarkers in US adults: NHANES 2003–06. Eur Heart J.

[10] Carson V, Wong SL, Winkler E, Healy GN, Colley RC, Tremblay MS (2014). Patterns of sedentary time and cardiometabolic risk among Canadian adults. Prev Med.

[11] Thorp Healy GN, Winkler E, Clark BK, Gardiner PA, Owen N, Dunstan DW (2012). Prolonged sedentary time and physical activity in workplace and non-work contexts: a cross-sectional study of office, customer service and call centre employees. Int J Behav Nutr Phys Act.

[12] Vandelanotte C, Duncan MJ, Short C, Rockloff M, Ronan K, Happell B, Di Milia L (2013). Associations between occupational indicators and total, work-based and leisure-time sitting: a cross-sectional study. BMC Public Health.

[13] Van Acker R, De Meester F, VIGeZ (2015). Langdurig zitten: dé uitdaging van de 21ste eeuw. Syntheserapport als actuele onderbouw voor de factsheet sedentair gedrag.

[14] De Cocker K, De Bourdeaudhuij I, Cardon G, Vandelanotte C (2015). Theory-driven, web-based, computer-tailored advice to reduce and interrupt sitting at work: development, feasibility and acceptability testing among employees. BMC Public Health.

[15] Ajzen I (1991). The theory of planned behavior. Organ Behav Hum Dec.

[16] Maes S, Karoly P (2005). Self-regulation assessment and intervention in physical health and illness: a review. Applied Psychol Int Rev.

[17] De Cocker K, De Bourdeaudhuij I, Cardon G, Vandelanotte C (2016). The effectiveness of a web-based computer-tailored intervention on workplace sitting: a randomized controlled trial. J Med Internet Res.

[18] Springvloet L, Lechner L, Candel MJ, de Vries H, Oenema A (2016). Exploring individual cognitions, self-regulation skills, and environmental-level factors as mediating variables of two versions of a web-based computer-tailored nutrition education intervention aimed at adults: a randomized controlled trial. Appetite.

[19] van Genugten L, van Empelen P, Oenema A (2014). Intervention use and action planning in a web-based computer-tailored weight management program for overweigth adults: randomized controlled trial. JMIR Research Protocols.

[20] Prapavessis H, Gaston A, DeJesus S (2015). The theory of planned behavior as a model for understanding sedentary behavior. Psychol Sport and Exerc.

[21] Gardner B, Smith L, Lorencatto F, Hamer M, Biddle SJ (2016). How to reduce sitting time? A review of behaviour change strategies used in sedentary behaviour reduction interventions among adults. Health Psychol Rev.

[22] Gollwitzer PM (1999). Implementation intentions: strong effects of simple plans. Am Psychol.

[23] Vallance JK, Courneya KS, Plotnikoff RC, Mackey JR (2008). Analyzing theoretical mechanisms of physical activity behavior change in breast cancer survivors: results from the activity promotion (ACTION) trial. Ann Behav Med.

[24] Chau JY, van der Ploeg HP, Dunn S, Kurko J, Bauman AE (2011). A tool for measuring workers’ sitting time by domain: the Workforce sitting questionnaire. Br J Sports Med.

[25] De Bourdeaudhuij I, Sallis JF (2002). Relative contribution of psychological determinants to the explanation of physical activity in three population based samples. Prev Med.

[26] Van Dyck D, Cardon G, Deforche B, Owen N, De Cocker K, Wijndaele K, De Bourdeaudhuij I (2011). Socio-demographic, psychosocial and home-environmental attributes associated with adults’ domestic screen time. BMC Public Health.

[27] De Cocker K, Duncan M, Short C, van Uffelen J, Vandelanotte C (2014). Patterns, correlates and moderating effects of occupational sitting in Australian employees. Prev Med.

[28] Craig CL, Marshall AL, Sjöström M, Bauman AE, Booth ML, Ainsworth BE, Pratt M, Ekelund U, Yngve A, Sallis JF, Oja P (2003). International physical activity questionnaire: 12-country reliability and validity. Med Sci Sports Exerc.

[29] Vandelanotte C, De Bourdeaudhuij I, Sallis J, Philippaerts R, Sjöström M (2005). Reliability and validity of a computerised and Dutch version of the International physical activity questionnaire (IPAQ). J Phys Act Health.

[30] Guidelines for data processing and analysis of the International Physical Activity Questionnaire (IPAQ)—Short and long forms. 2005. Retrieved from https://docs.google.com/viewer?a=v&pid=sites&srcid=ZGVmYXVsdGRvbWFpbnx0aGVpcGFxfGd4OjE0NDgxMDk3NDU1YWRlZTM. Accessed 19 Apr 2016.

[31] MacKinnon D, Fairchild A, Fritz M (2007). Mediation analysis. Annu Rev Psychol.

[32] Sobel ME, Leinhart S (1982). Asymptotic intervals for indirect effects in structural equations models. Sociological methodology.

[33] Storm V, Dörenkämper J, Reinwand DA, Wienert J, De Vries H, Lippke S (2016). Effectiveness of a web-based computer-taiored multiple-lifestyle intervention for people interested in reducing their cardiovascular risk: a randomized controlled trial. J Med Internet Res.

[34] de Vries H, Eggers SM, Bolman C (2013). The role of action planning and plan enactment for smoking cessation. BMC Public Health.

[35] Plaete J, De Bourdeaudhuij I, Verloigne M, Crombez G (2016). The use and evaluation of self-regulation techniques can predict health goal attainment in adults: an explorative study. Peer J.

[36] Koring M, Richert J, Parschau L, Ernsting A, Lippke S, Schwarzer R (2012). A combined planning and self-efficacy intervention to promote physical activity: a multiple mediation analysis. Psychol Health Med.

[37] Fuchs R, Seelig H, Göhner W, Burton NW, Brown WJ (2012). Cognitive mediation of intervention effects on physical exercise: causal models for the adoption and maintenance stage. Psychol Health.

[38] Jacobs A, Ammerman A, Ennet S, Campbell M, Tawney K, Aytur S, Marshal S, Will J, Rosamond W (2004). Effects of a tailored follow-up intervention on health behaviors, beliefs, and attitudes. J Women's Health.

[39] Oenema A, Brug J, Dijkstra A, de Weerdt J, de Vries H (2008). Efficacy and use of an internet-delivered computer-tailored lifestyle intervention, targeting saturated fat intake, physical activity and smoking cessation: a randomized controlled trial. Ann Behav Med.

[40] De Bourdeaudhuij I, Stevens V, Vandelanotte C, Brug J (2007). Evaluation of an interactive computer-tailored nutrition intervention in a real-life setting. Ann Behav Med.

