# Supplementary material for: What are the working mechanisms of a web-based workplace sitting intervention targeting psychosocial factors and action planning?
Source: BMC Public Health. 2017 May 3;17:382. doi: 10.1186/s12889-017-4325-5 (PMC5415713; doi:10.1186/s12889-017-4325-5)
Supplement: Supplementary file 2 — Questionnaire. Overview of the questions used in this study. (DOCX 14 kb) [file 12889_2017_4325_MOESM2_ESM.docx]

**What is your sex?**

Male/female

**How old are you?**

Open-ended (years)

**What is your highest educational degree?**

No diploma / Elementary school / Secondary school / High school / University

**What is the average amount of time you daily spent at work?**

Open-ended (hours and minutes)

**How long have you been working at your current employer now?**

Open-ended (number of years)

**What is your body mass?**

Open-ended (kg)

**What is your stature?**

Open-ended (m)

**How many time do you spent sitting while at work on a workday in the last 7 days?**

Open-ended (hours and minute per day)

**How many time do you spent sitting while at work on a non-workday in the last 7 days?**

Open-ended (hours and minute per day)

**To which degree do you agree with the following statements:**

1. **‘Prolonged daily sitting for long hours increases the risk of physical and mental health problems, like diabetes and depression’.**

Disagree/unsure/agree

1. **‘Even when one is being regularly active, i.e. daily walking, prolonged sitting increases the risk of physical and mental health problems’.**

Disagree/unsure/agree

1. ‘**It is healthy to interrupt periods of prolonged sitting, as the risk of health problem then decreases’.**

Disagree/unsure/agree

**To which degree do you agree with the following statements:**

**I think changing my sitting behaviour is…**

1. … **healthy.** Strongly disagree/disagree/neutral/agree/strongly agree
2. **… feasible.** Strongly disagree/disagree/neutral/agree/strongly agree
3. **… disturbing to others.** Strongly disagree/disagree/neutral/agree/strongly agree
4. **… awkward.** Strongly disagree/disagree/neutral/agree/strongly agree
5. **… relaxing.** Strongly disagree/disagree/neutral/agree/strongly agree
6. **… time-losing.** Strongly disagree/disagree/neutral/agree/strongly agree

**How certain are you about changing your sitting behaviour when…**

1. **… feeling tired/bad/tense/depressive?**

Strongly disagree/disagree/neutral/agree/strongly agree

1. **… when colleagues don’t do this?**

Strongly disagree/disagree/neutral/agree/strongly agree

1. **… when not being supported by your supervisors?**

Strongly disagree/disagree/neutral/agree/strongly agree

1. **… being busy or having high time pressures?**

Strongly disagree/disagree/neutral/agree/strongly agree

**Would your colleagues support you when trying to change your sitting behaviour?**

Strongly disagree/disagree/neutral/agree/strongly agree

**Are you intending to change your sitting behaviour?**

No / Yes, I may do this in the future / Yes, I will try this in the next weeks / Yes, I will start doing this right away

**During the last 7 days, on how many days did you walk for at least 10 minutes at a time?**

Open-ended (days per week)

**How much time did you usually spend walking on one of those days?**

Open-ended (hours and minute per day)

**During the last 7 days, on how many days did you do vigorous physical activities?**

Open-ended (days per week)

**How much time did you usually spend doing vigorous physical activities on one of those days?**

Open-ended (hours and minute per day)

**During the last 7 days, on how many days did you do moderate physical activities?**

Open-ended (days per week)

**How much time did you usually spend doing moderate physical activities on one of those days?**

Open-ended (hours and minute per day)
